# "The Hospital" Nursing Mirror

**Published:** 1900-02-10

**Authors:** 


### "The Hospital" Nursing Mirror.

Being the Nursing Section of "The Hospital."

[Contributions for this Section of "The Hospital" should be addressed to the Editor, The Hospital, 28 & 29, Southampton Street, Strand, London, W.C., and should have the word "Nursing" plainly written in left-hand top corner of the envelope.]

### Notes on News from the Nursing World.

### THE PRINCESS OF WALES'S WAR FUND.—THE RESPONSE OF THE NURSES.

The list of subscriptions to the Princess of Wales's War Fund, which was opened December 23rd, is now closed, the total amount realised from the nurses being £230 7s. 10d. This is a result on which we warmly congratulate them, especially those who so earnestly asked us to receive contributions. Bearing in mind the fact that the subscription was limited to 5s., and that a large proportion of the amounts subscribed were in smaller sums, the response to the appeal must be regarded as extremely satisfactory. Neither the solicitude of the nurses for the sick and wounded soldiers, nor their generosity in a good cause, needed to be put to the test; but they have once more shown how ready they are to bear their share, if not indeed more than their share, in any movement for the amelioration of suffering. We are sure that the Princess of Wales herself will fully appreciate the practical manner in which nurses from all parts of the country have manifested their desire to support her War Fund.

### NURSING IN MARITZBURG.—THE HORRORS OF WAR.

Our correspondent, who is nursing in a military hospital at Maritzburg, sends us some further notes:—"The heat is very trying, 135 deg. often outside, and 94 deg. in the wards; 88 deg. we think quite cool. Some of our cases are simply wonderful. I had Sir William MacCormac in two or three times to see some of the patients. I had one week a boy shot straight through the stomach, not a bit the worse; and others shot through in different places. At present I have one man shot through both lungs and liver. He is very ill, but will recover, I think. One I have for operation to-morrow, the bullet went in at his back and is to be taken out at the knee. It was shot straight down the leg. They are all X-rayed first. Sometimes we are in the theatre day and night after a rush, as we call it, and some of the cases are so sad. I am glad to be here, but never want to see another war. No one knows all the horrors of it who has not seen it. One poor sister had five deaths in her ward this week, principally head cases. We have a great deal of enteric owing to the bad water the men drink. A lady has taken a fine snapshot of my ward (24 beds) and of one of the tents. We have about 700 beds here; counting the other places in the town and Durban 2,000. We get the men well enough to travel here, take out the bullets, and then send them on. It is a constant change."

### NURSES AND THE STATIONARY HOSPITALS.

Sir William Thomson, in his address at the inspection of Lord Iveagh's Hospital by General Gosset, said, "Perhaps the only possible drawback is the want of female nurses. This, however, is not Lord Iveagh's fault, as the War Office authorities refuse to allow them to accompany the stationary hospitals. Only the base hospitals are allowed female nurses, the number being restricted to four for every 100 beds, despite the many volunteers who would only be too glad to offer their services. Indeed, the authority already mentioned states that beyond Cape Town there is not a single female nurse, with the exception of those locked up in Ladysmith, Kimberley, and Mafeking." We hear from a correspondent in South Africa that in one of these towns the nurses not only give all their time, attention, and sympathy to their patients—who are not, of course, always soldiers—but frequently buy luxuries at ruinous prices for them, occasionally even parting with their own clothes, though it is almost impossible to replace the latter. Lady Sarah Wilson, in the absence, we presume, of any trained nurses, has been placed in charge of the auxiliary hospital at Mafeking; and, notwithstanding that the Boers persistently shell it, she has so far escaped injury.

### NURSING "TOMMY" AT WYNBERG HOSPITAL.

An interesting account is given by Sister A. B. Smith, who is at present engaged in nursing at Wynberg Hospital, in a letter to her brother, the rector of Middleham. Miss Smith, writing on the 8th of last month, says:—

"I have a great many Highlanders among my patients; some that were left of the Black Watch, and some Gordons and Seaforths. You would be amazed at the way some of these men are positively riddled with shots and get well. On the whole, the bullet wounds do remarkably well. I think I am most sorry for the men who come down sick with dysentery and typhoid fever; they feel it frightfully, and would much rather be wounded; they look on it as a disgrace to be ill and not wounded. I am at present nursing 'Tommy,' not the 'officer,' with one Boer patient—such a nice man, who comes from Johannesburg. The men are all very interesting to talk to. They tell me the most awful part of it all is lying on the field after being wounded, and fearing they won't be found at all. Sometimes it is as long as 48 hours before they are discovered. The majority of them are only anxious to get well and go to the front again. I had 60 patients to look after, but now some have gone away. The situation is perfect for a hospital. There are lovely woods all around, and the climate is charming. The people in the neighbourhood are most generous in sending good things for the men, and every day I get fresh milk, eggs, jellies, custards, &c. We have a nephew of General Wauchope's here very severely wounded, but he is going to get well. We have also a Boer officer named Pretorius, who has been obliged to have his leg amputated. He says he is very sorry for us, but we are to be beaten!"

### THE NURSES OF THE SEVENTH GENERAL HOSPITAL.

In accordance with the usual custom only nine female nurses have been appointed to the Seventh General Hospital, including the superintendent, Miss F. E. A. Williams, of the Army Nursing Service. The other sisters of the Army Nursing Reserve are the following: Miss Louisa Basan, who was trained at St. Bartholomew's Hospital, afterwards serving at "Bart's" as staff nurse and as a member of the Nursing Institute, and subsequently as charge nurse and night superintendent at the Fountain Hospital, Tooting; Miss S. C. Chown, who was trained at Portsmouth Hospital, where she was subsequently staff nurse and sister, and has since been engaged in private nursing; Miss A. N. Ferguson, who was trained at the Longmore Hospital for Incurables, Edinburgh, and the County Hospital, has since been district nurse at Ancoats and Settle, ward nurse and sister at St. George's Hospital, and secretary of the Chartered Nurses, Princes Street, Cavendish Square, London; Miss M. C. McNeill, who was trained at the Royal Hospital, Belfast, where she was staff nurse until October 6th, and has since been engaged in parish nursing; Miss L. M. Fletcher, Miss E. M. Gardner, Miss M. L. Gordon, and Miss E. H. Wilson.

### NURSING IN AN AMBULANCE TRAIN AT THE WAR.

MRS. BAGOT, who went to South Africa in connection with the Portland Hospital, has sent home an account of an ambulance train which she saw before it started for Modder River. She says:—

"It is charmingly arranged and thought out; nothing could surpass the comfort, with excellent kitchen, pantry, and dispensary on board. There was room for about 100 men, a passage running down the train with a double row of bunks on either side. Most of the wounded men are brought straight off the battlefield, and carefully washed and put to bed at once. Of course, in many cases it is wiser not to remove the field dressings in the train, but occasionally even operations are performed when necessary. A complete kit of shirt, sponge, brush, socks, handkerchief is in readiness for every man, with knife, spoon and fork and cup within reach; the bunks are so arranged that the sides can be taken away to allow each patient to be removed from the stretcher without the slightest discomfort. Besides the surgeons there are two nursing sisters and a good number of carefully trained orderlies. But the anxiety of the journey is terrific; one sister said she had never once laid down for four nights and days when they were bringing wounded down. She told us the men always entreated to be washed before they would eat any food—after weeks and weeks without water this is not surprising. Her account of the journey from Magersfontein was heartrending, for the Highlanders' grief over their reverse was terrible."

At the time of writing, Mrs. Bagot was not able to report that the Portland Hospital was in the midst of its work for the sick and wounded.

### ARMY NURSES IN AMERICA.

AT the commencement of the Spanish War only American citizens were accepted as army nurses, but, as the demand became greater, applicants were engaged irrespective of nationality. The number of applicants has, however, since become so large that the Surgeon-General has decided that only nurses who have lived in the United States for at least ten years shall be eligible for original appointments.

### ST. GEORGE'S INFIRMARY.—CONCESSIONS TO NURSES.—RESIGNATION OF THE MATRON.

AFTER all, we are glad to learn, the Visiting Committee of St. George's Infirmary, Fulham Road, have at length decided upon some reforms. On Friday last a special meeting of the committee took place, and it was resolved to make additions to the nursing staff and to increase the off-duty time. At the meeting the statements of our correspondent were further considered, but while the nurses are to benefit in consequence of her charges, nothing is to be done for the patients. The committee continue to defend the system of nursing children and adults as "the best arrangement possible," though a visitor—not our correspondent—says that she has "often seen rough girls and women striking little children;" and our correspondent herself lately saw "a girl of very bad character, in the ward with children, nearly shake the breath out of a very feeble old woman who was a little out of her mind." The question of the patients' food, and the long interval between the meals, was discussed at the special meeting, but the committee again declined to take any action. Meanwhile, we hear that the matron has resigned. Possibly a new matron will be both willing and able to insist upon the proper treatment of the patients. The small concessions to the nurses is a step in the right direction, but it will not silence the demand for reform in the interests of those who are less capable than the nurses of helping themselves.

### THE AGE LIMIT IN NURSING.

THE contribution by a matron of experience in our issue of last week on the "Commercial Element in Nursing," is followed this week by an article on the age limit from the pen of Miss Mary Gardner, lady superintendent of the Birmingham and Midland Counties Sanatorium. It is always an advantage when a contributor, especially one whose practical knowledge invests her opinions with weight, allows her name to be used. Miss Gardner's paper on the age limit would, however, in any case have merited attention. It constitutes a very powerful, and indeed, crushing, indictment against the policy of excluding anyone from nursing on account of her age. The only test of value is capacity, and so long as a woman is physically and mentally able to discharge her duties she should not be disqualified by age for any position in the nursing world. It is obvious that while comparative youth often means comparative ignorance a woman of more mature years enjoys all the inestimable advantages of ripe experience.

### CHOOSING A NURSE FOR HER LOOKS.

THE Belfast Board of Guardians have been appointing five temporary probationers. The *Irish News*, in its account of the proceedings at the meeting, reports that after Mr. Allison had proposed the appointment of Miss Gold Mr. Oswald said, "I think it is agreed that she is the best-looking girl in the crowd." At a later stage of the meeting, after "the best looking girl in the crowd" had been appointed, she returned thanks—the report says—in felicitous terms, "and on leaving the room was made the recipient of prolonged applause amid much laughter." As she retired, Mr. Sinclair observed, "That takes the cake away," whereupon there was renewed laughter. This is the sort of procedure that tends to make a Board of Guardians a by-word in the country.

### A NURSING ASSOCIATION IN NEED.

ALTHOUGH it was the unpleasant duty of the chairman of the Ashton and District Sick Nursing Association, at the annual meeting, to announce a deficit on the year's working, the balance on the wrong side is so small that there ought not to be the slightest difficulty in putting things right. It was agreed by every speaker that the association is very economically managed, and cordial testimony was given to the manner in which the lady superintendent and the staff nurses discharge their duties. The unsatisfactory feature is that "the great mass of the public" do not support the society as it deserves. This is the more reprehensible because Ashton is a centre of mill-life, and in cases of illness trained nurses are particularly wanted. In order to secure their services the employés at the mills should make a point of subscribing regularly a small sum to the Nursing Association, which only needs sufficient supply of their pennies to keep it going on its present basis.

### ENTERTAINMENT BY NURSES AT PARK HOSPITAL, HITHER GREEN.

On Wednesday and Thursday last week an excellent entertainment was promoted by the nurses of Park Hospital. The room in the Nurses' Home was tastefully decorated. A bijou stage with electric footlights was erected, and an amusing and patriotic programme was gone through. An opening song, "The Pirate of Penzance," was rendered by a chorus of nurses, followed by Miss I. M. Thomas in the popular recitation, "An Absent-minded Beggar." This was delivered so admirably that it provoked cheer after cheer, an indication that the nurses are following the events of the day with great interest. Nurse Archbold sang, in costume, "Follow On," with chorus by the nurses, which was very well received. A dialogue by Nurse Phillipault was very funny. Nurse Archbold was encored for her next song in costume, "The Soldiers in the Park," and then gave a tambourine dance well worthy of the other profession. The entire company sang two songs, "The Purity Brigade" and "Soldiers of the Queen," the chorus of the latter being joined in by the whole of the audience with enthusiasm. Dr. Adkins sang "Yuss," by Chevalier, and, being encored, gave "A Jovial Monk am I." The entertainment concluded a pleasant evening with a farce, "Difficult to Please," by Nurses Oliver, Archbold, and A. Rangel. The staff afterwards had a dance.

### THE INTERNATIONAL HOSPITAL AT NAPLES.

Some further particulars of the International Hospital at Naples have been sent to us from an authoritative source. It was founded in 1877 by foreign residents to receive sick foreigners without distinction of country or of creed. In 1884 it was transferred from the town to its present situation, a large villa high up in the best quarter of Naples, with a fine garden and three large terraces, commanding lovely views. This villa is called after Lady Harriet Bentinck, who, dying in Naples, bequeathed the sum of £4,000 sterling for its purchase, about a similar sum being expended in additional wings and improvements. There are larger wards for gratis and third-class patients, and separate rooms for private patients, paying according to their means. The total number of beds is 32, but they are very rarely all occupied at once. A large proportion of third-class patients are British sailors. Those belonging to merchant vessels subscribing to the hospital are taken free, while those of the navy are paid for by the British Government. The greater number of paying patients are admitted in February, March, and April, which comprise the so-called Neapolitan season; and many persons from various portions of the globe who experienced the discomfort of falling ill in a Neapolitan hotel have fully appreciated their treatment in the International Hospital, and year after year send subscriptions to enable it to help others in their need. As the season is so short the patients' payments naturally only cover a minimum part of the expenditure; and though the donations of the residents and visitors furnish a larger sum, there is almost always at the end of the year a considerable deficit to be met. All Consuls have the right to send for gratis treatment indigent cases, and this privilege can also be exercised in favour of an applicant by any two members of the committee. The Dutch Consul is president, the British Consul is vice-president, and the committee consists of 15 leading citizens of different nationalities. The house surgeon is German, the matron English, both having a conversational knowledge of English, French, and German. The nurses must have received a thorough training in a general hospital. They are usually English or German, and speak both languages. No probationers are received. Patients may call in for consultation any medical man they desire, and may choose their own surgeon for operations. The hospital has been visited by the Princess of Wales, Princess Victoria of Wales, the Empress Frederick, the Princesses Victoria and Margaret of Prussia, by many medical men, and matrons of English and American hospitals, who have expressed themselves pleased with its lovely situation and general arrangements for the treatment and comfort of the patients.

### NURSING AT BILLINGSGATE.

We are glad to hear of a useful work in connection with the Billingsgate Christian Mission. There is, it seems, a first aid branch. This is under the charge of an experienced nurse, resident on the premises, and is for the purpose of rendering first aid in all cases of accident, for dressing wounds, &c. The City Corporation have generously defrayed the cost of an ambulance of the most improved pattern for the purpose of removing bad accidents to the hospital. Considering that there are over 3,000 porters employed in the fish markets and at the adjacent fruit wharves, besides men off the steamboats, fishermen, van men, &c., there are almost necessarily many casualties. To go to hospital often means the loss of two or three hours from work, and some of the fishermen would not have as much time on land. Working among fish entails great risk of blood poisoning by the slightest wound, therefore prompt treatment is of the utmost importance, and it is certain that immediate careful antiseptic dressing has in many cases saved a limb. The work can scarcely fail to increase as it becomes more widely known. The men appear to appreciate it highly. "Why have you taken off your bandage?" asked the nurse of a patient who had been neatly dressed the day before. "Please, Sister, I put it in my pocket to keep it clean," was the answer. But, as a rule, they are most obedient to the instructions not to interfere with their dressings. The fish market is open at five a.m., but as the nurse cannot be on duty night and day, she begins at seven a.m.

### SHORT ITEMS.

At the annual meeting of the Glasgow Maternity Hospital the other day testimony was given to the value of the work done by the 120 nurses who are under the charge of the admirable matron. The chairman stated that there were always more nurses desirous of coming in than there were vacancies.—Miss Rose Petty has been appointed by the London School Board an inspector of the homes in which blind and deaf children are boarded out. Miss Petty was trained at the London Hospital, and possesses the Sanitary Institute certificate. For the last 16 months she has been lecturing on health for the School Board.

### Lectures to Nurses.

By CARSTAIRS DOUGLAS, M.D., B.Sc.Edin., Professor of Medical Jurisprudence, Anderson's College Medical School, and Dispensary Physician, Samaritan Hospital, Glasgow.

### DIETETICS AND THE PHYSIOLOGY OF DIGESTION (*continued*).

In this, the concluding lecture of our series, we shall devote ourselves to the consideration of dietetics proper, that is to say, the selection of food stuffs in proper proportions and of a nature suitable to the needs of the person under observation. Now to take the case of an ordinary individual in health, doing a moderate amount of work, the question of feeding is found to rest on a definite principle, viz., that as much of the various elements, carbon, nitrogen, oxygen, &c., must be taken in each day as the body disposes of, if the equilibrium of weight is to be maintained. And an analysis of the excreta reveals this fact, that such a person throws off from the body every twenty-four hours about 250 grammes of carbon (one gramme is equal to about 15½ grains) and about 16 grammes of nitrogen. It is obvious, therefore, that to supply this loss, an equivalent amount of these elements must be supplied each day in the food; and it will also be noted that the amount of carbon greatly exceeds that of nitrogen, in the proportion of 15½ to 1. An ideal dietary must therefore not only yield the requisite amounts of carbon and nitrogen, but must yield them in the proper proportions, and thus it is that for man a mixed dietary is practically essential. We find that the most suitable dietary is one where sufficient proteid is present to give us our 16 grammes of nitrogen, while the balance of the carbon is supplied by carbo-hydrates and fats. Moleschott suggests the following combination: Proteid, 120 grammes; fat, 90 grammes; carbo-hydrates, 333 grammes. Such a diet would yield the proper amount of carbon and of nitrogen and in the proper proportions, and might be made up of lean meat with a little fat, bread, butter, potatos, porridge, and milk.

In the case of a sick person, however, confined absolutely to bed and exerting himself to the least possible degree, not only is much less food required, but it does not matter so much if for a time the above ratios are not strictly observed. We find accordingly that milk is a very suitable food in such cases; it contains all the requisite food elements, and although the proportion of carbon to nitrogen in it is about 10 to 1 (showing a relative poverty in carbon), this does not matter much, since the patient is developing comparatively little bodily heat and energy, for which carbo-hydrates and fats are essential. When milk forms the sole food, an adequate quantity must be allowed, say a minimum of two and a-half pints per diem, and rather more than this (three and a-half to four pints) if the patient can digest it. Milk should be given diluted with ordinary warm water, lime-water, potash, soda, or Apollinaris water, or with barley-water. It may be pancreatised or peptonised before administration if the digestion be very feeble, and it is often advisable that it be sterilised before use, which can be accomplished by keeping it for twenty minutes at a temperature of 160 deg. Fahr. In all cases of pure milk dieting, the nurse should examine the motions to see that the patient is not passing masses of undigested curd. Milk is our sheet anchor in enteric fever, and is also of great value in most febrile conditions, in acute and chronic gastric catarrh, in gastric ulcer, and in many other morbid conditions. It should, as a rule, be given in definite quantities, say two to six ounces, every two hours.

In beef tea we have a food of considerable service in the sick-room, and one which, on account of its stimulating properties, often forms a valuable adjunct to a milk diet. It is a food that has often been condemned as being merely a solution of meat extractives, such as creatinin and urea and various salts. As a matter of fact, beef tea properly made contains a good deal of albuminous material, and notably of gelatine. We saw that gelatine was capable of being digested and converted into peptones, and it is thus able to supply nitrogen to the tissues and to save them from wasting so much in disease as they otherwise would; to use the phrase of the French writers on dietetics, gelatine acts as a "*moyen d'épargne*" for the tissues.

We may here discuss the question, Is alcohol a food or a medicine? I think we may say it is both. In moderate doses most of the alcohol taken is burnt off or consumed in the body; by its oxidation it therefore saves other tissues, such as fat and proteids, from waste by combustion. As a drug it stimulates the action of the heart and large vessels, leads to dilatation of the skin vessels (and so to loss of body heat), and also quickens the digestive processes. It is often of great value in febrile conditions with exhaustion, such as pneumonia, septicæmia, typhoid fever, &c.

In gastric ulcer we have a disease where the nurse must exercise great care in feeding. In any acute case of gastric ulcer, with, let us say, pain and some tendency to bleeding, the patient should be absolutely confined to bed and put on a complete milk diet. Two ounces of milk diluted with lime water may be given every two hours at first, the amount being gradually increased. The bowels may be emptied by enemata for the first few days, and thereafter a dose of Carlsbad salts may be given every alternate morning. As the stomach improves Benger's food and chicken-tea may be added to the dietary, and later on porridge made with oat-meal flour, rusks prepared with boiled milk, and veal or mutton broth. After a suitable time the patient may get a little steamed fish, and thus the way may be paved for the ingestion and digestion of stronger foods.

In enteric fever feeding is of paramount importance. Milk diet pure and simple, if it can be borne, is the best of all. Fifty to sixty ounces, suitably diluted with plain, barley, or lime water, may be given in twenty-four hours, a measured quantity being allowed every two hours. If the digestion is weak the milk may be partially peptonised first. If it disagree, or if it be considered advisable to supplement it, a little well-strained chicken tea may be permitted, or albumin water, made by straining the raw whites of eggs through muslin, and diluting with an equal amount of water; this may be flavoured in any suitable way.

Diabetes mellitus, to take another example, is a disease which, for its amelioration, depends more on diet than on anything else. Since in it a large quantity of grape sugar is thrown off by the kidneys, it is essential that the food chosen be such as will not favour the manufacture of sugar within the system. All saccharine and starchy foods must therefore be forbidden as far as possible, and the diet made almost wholly proteid. Even with a proteid diet a certain amount of sugar is formed, and this diet may therefore be eked out advantageously by the addition of fat. All diabetics may be allowed a good supply of butter, there being no certain proof that sugars are ever formed from fats within the system.

In bringing this lecture to a close I feel that some apology is necessary for its sketchy and fragmentary character. It is, however, impossible in such a limited space to do more than merely touch on the fringe of the subject. I trust, however, that enough has been said to make clear and simple the physiology of digestion, and to awaken in the minds of my readers some real interest in the important subject of dietetics.

### The Queen's Visit to Italy.

### NURSING IN BORDIGHERA.

Sitting on a wide white loggia, lifted high above the olive gardens beneath into the sparkling air, I wish my brain was a sensitised plate to record the impressions before me. Eastward, on the higher ground of the promontory, its yellow houses and red roofs sharply silhouetted against the sky, stands the little walled town of Bordighera. Westward, over the French borderland, less than twenty miles away, and in abrupt contrast to the soft outlines of the nearer volcanic hills, I watch the ridges of the Maritime Alps grow purple in the early sunlight, while lying at their feet, wrapped in a gossamer veil of mist, I distinguish Mentone in its sheltered bay, with Monaco and Monte Carlo beyond. Palm gardens and olive groves, and plantations of roses and carnations cover the plain below and stretch over the hillsides and downward to the sea, broken only by the gleaming of villa walls and the green domes of pines. Till comparatively recent years little was known of Bordighera by the traveller. Then the appearance of Ruffini's novel of "Dr. Antonio" drew attention to its gracious charm, and winter after winter and spring after spring saw the same wanderers return again, finally to build themselves homes amid the olive woods of the hillside. Now the proposed visit of the Queen of England confers a last distinction upon the little town, and surely this region of roses, of sunlit valleys folded among the hills, of sunsets whose splendours over sea and sky recall those of Venice, is worthy of this Royal favour. Looking down the Strada Romana below I see a gable of the Hotel Angst, which the Queen is expected to occupy, and already the Bordigheresi are looking forward to the spring with lively interest. Considered from the climatic point of view the place possesses one or two special features, for the sun is said to shine here two hours longer than on any other Riviera station, while the air has a peculiarly saline bracing quality.

Gleaning all I can of professional interest, as it is the journeying nurse's hobby to do, I learnt that the needs of the sick poor are now met by a recently-established "Casa Providenza," and, therefore, set out at the first opportunity on a voyage of discovery. Toiling up the stairway-street by which the mouldering outer wall of the old town is approached, I stood for a moment to look down its steep perspective to the shining sea below, and note the quaint framing of the picture by the arched gateway of the Porta Sottana. Here and there above my head a high-set window was ingeniously festooned with the family washing, while from another hung a tangle of geranium in full flower. Within the gate I found myself in a mysterious labyrinth of narrow lanes and flights of steps disappearing into shadow—of sombre walls, with archways connecting the houses high above the street to give security in case of earthquake. Dark eyes watched me curiously from open doorways, till finally, guided by a Bordigherese maiden, I emerged into a cheerful piazza with a running fountain. Here stood the church, and opposite it, the object of my search, maintained, as the legend over the door informed me, by private charity. A smiling Italian face appeared in answer to my ring, and on asking for the Suore and explaining my errand, I was kindly welcomed by the Mother Superior and her one assistant nurse, "Daughters of Saint Anna." They had only four patients in the house, they told me, visited by an English doctor in charge, but it would give them infinite pleasure to display their arrangements to me. So, talking volubly all the way, they took me up a flight of narrow but well-kept marble stairs to the first floor. Here were five good-sized whitewashed rooms, with a crucifix, a single bed, and a minute washstand in each. Other beds could, of course, be added in case of need, the sisters said, displaying these absolutely bare but not uncheerful apartments with much pride. The patients, of all nationalities, were generally workmen who had received hurts in building, while one bed was always reserved for aged chronics. Another well-lighted room was shown me as the "Camera antisettica" for operations, but as it was perfectly empty, and guiltless of any trace of equipment, I presumed that the surgeon probably brought with him all that was necessary. I was introduced cordially to each patient, all of them sitting up in solitary state in their rooms. One was a young foreigner from the Hotel Angst, another a workman with a broken arm in a homely bandage. With the eighty-year-old "Vecchia" I made fast friends, and was amused to find the worthy lady capable of executing a little pirouette on receiving a trifling gift. Finally, Guiseppina, aged two, a foundling baby, and evidently the darling of the good Sisters' hearts, was presented to me, and then, Guiseppina accompanying, we proceeded downstairs again to the lower regions. Here I saw the low-ceiled kitchen, "where the Sisters had their meals," and beside it a sort of scullery, where the poor to whom the priest has given tickets are warmed and fed, while two other rooms, the one with two, the other with three clean beds, received any homeless wayfarers who begged a shelter. These carefully-kept guest chambers recalled the tender charities of St. Francis to my mind as I looked at the crucifix upon the walls. Primitive, indeed, and simple was every arrangement; rules and ordinances, as we nurses understand them, seemingly unknown, though the house combined the functions of hospital and soup kitchen, foundling home and casualty ward in one. But a cheerful, happy spirit seemed to rule the minds of the "Daughters of Saint Anna," and I parted from them, and Guiseppina, at the door with a very friendly feeling. It was growing late, and the whole population seemed in the streets, for the men had come home from the hill-sides, and the children from gathering in the goats. My last glimpse of the groups in the Piazza was a very animated one, and when I emerged from the town into the silence without the gate I seemed to have come into another world. Far below lay the sea at high tide, "too full for sound or foam," and as I stood for a moment on the hill the bells of the campanile began suddenly to ring the "Ave Maria," and a single pale star pierced the yellow sky.

### Appointments.

Porthcawle Rest Convalescent Home, Glamorganshire.—Miss Ethel Gray has been appointed Lady Superintendent. She was trained at Edinburgh Royal Infirmary, and has been matron of Ulverstone Hospital.

Home of St. Barnabas, Lingfield.—Miss Mary Nicholson has been appointed Matron. She was trained at the Queen's Hospital, Birmingham, for three years. Her next experience was at the Aberdeen Royal Infirmary, where she was head of the female wards. Miss Nicholson has since been engaged in private nursing.

### To Nurses.

We invite contributions from any of our readers, and shall be glad to pay for "Notes on News from the Nursing World," or for articles describing nursing experiences, or dealing with any nursing question from an original point of view. The minimum payment for contributions is 5s., but we welcome interesting contributions of a column, or a page, in length. It may be added that notices of entertainments, presentations, and deaths are not paid for, but, of course, we are always glad to receive them. All rejected manuscripts are returned in due course, and all payments for manuscripts used are made as early as possible at the beginning of each quarter.

### The Age Limit and the Commercial Element in Nursing.

By MARY GARDNER, Lady Superintendent Birmingham and Midland Counties Sanatorium.

IN a recent issue of THE HOSPITAL "Nursing Mirror" it was reported that a nurse had been called upon to resign by the Scarborough Board of Guardians on being found to have understated her age by six years. Without knowing all the circumstances it is impossible to pass judgment on the case, but if there were no other cause for dismissal but the deception the sentence would appear one of rigorous justice, untempered by mercy. One is impelled to ask, Would the Board have engaged the nurse if she had given her true age? And if not, did they realise that she was under an overwhelming temptation to tell an untruth?

The incident is suggestive as a sign of the times. The growth of the "commercial element" in nursing, lately deprecated by a leader of the profession, is due to the very efforts, laudable in themselves, which have been made to systematise the art of nursing and place it on a professional basis. Now that the work has lost, or nearly so, its religious and voluntary character, and become an affair of exchange and barter—so much training and professional skill, so much wage—it is subject to the same laws of supply and demand which govern the general labour market, where youth is at a premium.

### THE STRUGGLE FOR EXISTENCE.

Nothing is more sad in the present keen struggle for existence than the thrusting aside in favour of younger competitors those men and women who have passed their youth. To appear old is fatal. Therefore, since these workers must live, for few have the courage or resignation to die for abstract principle, they resort to deception, if possible. It has been stated that the largest consumers of hair-dye are not fashionable women who wish to hide the traces of time, but labouring men, who are painfully aware that grey hairs will debar them from employment, to whom work is daily bread—in the majority of cases not for themselves only. Many who might singly give up the battle and lay down their arms in the spirit of the dying weaver in Mrs. Gaskell's "Mary Barton," "My God! I thank thee that the hard struggle of living is over," have families dependent upon them for whose sakes they dare not surrender. For it is not only the old, those whose working day may fitly be considered over, who are thus cast adrift, but men and women in middle life. The bitter cry of the supplanted grows more intense. Recently I read an announcement in the press that it was proposed to form an association on behalf of those who were considered "too old at forty"—which age seems to be the Rubicon. Too old at forty!—with life's race only half run! It is pitiable, pathetic, heart-breaking. . . .

### THE PROBLEM TO BE FACED.

We have to face the problem: What is to become of the workers of forty years of age and upwards? They cannot be allowed to starve. If they cannot support themselves they must be supported by others, even if it be in that last refuge of the destitute, the workhouse. We shall suffer as a community by the ruthless crushing out of the older workers. In a strong word picture Ruskin has satirised the folly of two rival shopkeepers who strive by close competition to ruin each other, which he terms fighting for the privilege of maintaining the other with his wife and family in the workhouse. The Board of Guardians or any other employers, corporate or individual, who reject or dismiss an employé as "too old" may be responsible for condemning such an one to subsistence on the rates, no impossible or remote contingency, even in the case of nurses or others of superior rank in life. In my personal experience I have met with not a few men and women of good birth and former station in the workhouse. But if a thought of such consequence were entertained it might be accompanied by the selfish reflection: "Such an one is not of this parish. The burden will not fall upon us." Thus we go on blindly, doing wrong, inflicting injury, because the consequence does not affect us directly, and we do not see or feel the ultimate result of our actions. But the retribution is sure. It is a truism that we suffer or gain through each other. The welfare of the community is the welfare of the individual. We must look for remedy in this matter to the united action of employers, for which end we need a more enlightened and liberal public opinion—more recognition of the claims of the older workers, of their right to live and work, and of the fatal short-sightedness of condemning them to the grim alternative of dependence or want.

### HOW WOMEN SUFFER.

Men suffer severely through the age limit, but women, as individual workers, suffer more widely from the same cause. Their working day is held to be shorter, while the average expectation of life is longer and their wages are lower. Moreover, advancing years bring less universal disadvantage to men. For them there are highly-skilled callings in which age and experience are allowed to carry weight, *e.g.*, the medical profession, of which the female members are so greatly in a minority that it may be considered for the purpose of this argument as a masculine profession. Years and grey hairs, presupposing accumulated knowledge and experience, tell in favour of the physician or surgeon if he has won a reputation and is not actually decrepit; but how different the case in the nursing profession!

### THE NURSING PROFESSION.

Although the nurse must begin her training some years later than the medical student (and few who know all that nursing involves advocate an earlier beginning than is at present the rule), she reaches the zenith of her career from thirty to thirty-five. After thirty-five the period of anxiety begins for her, increasing and intensifying year by year as she approaches the end of the thirties. Unless securely established she hardly dares to confess to thirty-nine, and forty sounds like the knell of her hopes and prospects. Yet she may be in reality in her prime, conscious of unimpaired activity, and capacity improved by experience. The cruelty is apparent. If, feeling keenly the hardship of her position—and more or less vaguely, perhaps, a sense of injustice, with the prospect of from thirty to forty years of life before her, during which she must work in order to live and provide for veritable old age—she states her age as less than the reality, dare those who have never been tempted to similar falsehoods condemn her harshly?

### A PERSONAL EXPERIENCE.

Some years ago I was acting as deputy matron in a provincial hospital during an interregnum, when a ward sister's post became vacant. Among the applicants was one in whose favour I was strongly prepossessed. Her training and qualifications, as shown by her certificates and testimonials, were all that could be desired. Her age was given as thirty-six, probably a strictly truthful statement, borne out by the date of training and length of subsequent work, also by her photograph, which was very pleasing, showing an intelligent and good face, indicative of a fine character. One could readily believe the original to be the high-principled, conscientious woman she was said to be—one, therefore, who would not deviate from the truth. But when her application was submitted to the chairman of the nursing committee he promptly rejected it on the ground that she was "too old." I ventured to appeal against this decision, the hardship of which, although then a young woman, I felt keenly. I reminded the chairman, who was also the senior staff surgeon—a man of at least fifty, but in the prime of vigour, who would doubtless have been astounded at a suggestion that he was past work, or even that he was not at his best—that a nurse cannot begin training before twenty-three or in some hospitals twenty-five, and three years are spent in training. She must therefore be at least twenty-six before she is acknowledged as a trained nurse. Was it just after so long and deferred a training that she should be set aside at thirty-six? The appeal was useless. "We would rather have a younger woman," was the only response. "But what are women to do after thirty-six?" I urged. "Oh, let them get married," was the answer, spoken in jest, but the refusal was in earnest. I fear no thought was given as to what should in reality become of women workers after thirty-six years of age. The incident made a deep impression upon me. I have since met with much corroborative experience.

### The Modern Nurse.

In the early days of trained nursing the conditions of work were so severe, entailing such long hours and laborious service as to tend to eliminate all but the physically strongest. Many a valuable life has been sacrificed, witness the death at thirty-six from sheer overwork of that brave pioneer of workhouse nursing reform, Agnes Jones. But in the present day the conditions are enormously improved. We have well-staffed hospitals, consequently shorter hours and more liberal leave and vacation. There is, therefore, no danger of a nurse being worn out at thirty-six or even forty years of age if of good constitution. She may have lost something of early freshness and physical energy at forty, for nursing still "takes it out of you," as the saying is; but if made of the right stuff she will have gained more than she has lost. Nursing is not simply manual work. Physical strength and activity are necessary, but mental and moral qualifications are even more indispensable. It is work which calls for the exercise in more or less degree of all that is best and highest in womanhood. The ideal nurse should be "a perfect woman nobly planned," but we usually have to accept less than perfection. Nevertheless there are many noble women who are also admirable nurses in our ranks, but they are nurses by virtue of training, and that training is not rounded off and completed by the three years' curriculum, and sealed by its certificate. All nurses live to learn the truth of the axiom that the art of nursing is never learnt. Experience in mere professional detail is as essential to the nurse as to the doctor, but further, there is no work in which personal character tells for more. Patience, tact, sympathy, the power to inspire confidence, to influence and control are not conspicuously the attributes of youth, but where existing rudimentarily are developed and perfected by time and exercise. How far the more strictly mental faculties of memory, observation, forethought, accuracy, &c., may be called out and developed by practice, we, who have watched the transmutation of the raw material into the finished product of trained nursing, know; but the process does not end there.

### The Evolution of the Matron.

But if the evolution of the highly trained nurse be slow, what shall be said of the matron? A matron must be a nurse and much more. It is not my purpose to enlarge here on all that that "much more" implies. It will be conceded that the office of matron is a responsisible and onerous one, affording scope for the exercise of talents of no mean order. After the preliminary training, the future matron must pass by successive steps through the different grades of her profession—staff nurse, sister, superintendent, or assistant matron, lastly, as a rule, matron of a small hospital, before she is appointed to a large one. But when thus qualified for the highest posts in her profession, how few years elapse, far less than are spent in the necessary preparation, before she is considered too old! In modern advertisements the age limit is generally from thirty to forty, and frequently the superior limit is fixed at thirty-five. Even if it is not so, the matron who owns to forty years of age finds her chances of an appointment seriously diminished. Again the question arises, What are matrons, who have spent years in training for their work, to do after thirty-five? It is not always possible or desirable in their own or other interests to hold the same appointment from thirty-five until able to retire, which, if a woman has no private means, but is dependent upon what she can save, she can rarely do before sixty. Nor as a rule does she lose interest in her work or desire to give it up earlier.

### The Age Limit in Operation.

I have lately seen two advertisements to this effect: "Wanted, a matron. Age from 25 to 35." One may pity the hospital presided over by the matron of 25, whose training must necessarily be incomplete and her experience nil. But such advertisements are, fortunately, exceptional. The lower limit is usually thirty, and thus the period of selection is brought within very narrow boundaries. Yet, excluding those cases, not impossible, where a candidate is appointed for her personal attractions—in which, of course, the younger women have the advantage—we must believe that committees as a rule have the interests of their institutions at heart; and are convinced, therefore, that they consult these best by selecting a woman from thirty to thirty-five. But do they always make the best choice when so limited? I venture to think not. The qualities which go to make a successful administrator and ruler over others are of slow and late development. If a matron is good at thirty she will be better at forty. The statesman of forty is but a stripling, and the ideal matron should possess—if I may so express it—statesmanlike qualities. Space forbids enlarging on this topic, but it is indisputable that the powers of organisation and government are developed and increased by experience.

### An Objection Answered.

Probably the cause of some of the reluctance which is shown to appoint a woman of forty lies in the fear which is felt of the physiological crisis through which women pass between forty and fifty. But inasmuch as it is a natural, not a pathological, change, it need not be feared for the normally healthy woman. Doubtless it is more often pathological than it need be, and doctors see the worst in this respect; but I believe that an exaggerated dread exists, and that it would be safe to say that in the great majority of cases the crisis is passed with little or no constitutional disturbance. And when over there is often a renewal of vitality for the woman, a rejuvenescence to which there is nothing in men's lives to compare.

### An Appeal to Men.

I should be sorry if any words of mine were interpreted as a defence of untruth in the matter of age or anything else. Such falsehood is degrading to women even when it seems forced upon them. For the sake of our self-respect, therefore, I wish that truth were made a less heroic virtue. I appeal to men, who find life's struggle hard for themselves, not to make it unnecessarily hard for women, who are more heavily handicapped by nature, and especially to medical men, not to condemn as "too old" nurses, their colleagues, at an age when they themselves are just beginning to gather in the harvest of their experience.

### An Experience in the Sierra Nevada Mountains.

By a Nurse in America.

Accustomed as I am to obey various duty calls, I must own that I felt greatly inclined to shirk one that I had lately. I was roused one night and told I was wanted to go to "Camp Badger," in the Sierra Nevada Mountains, to nurse a surgical case. It was not so much the locality—a once noted resort for train robbers and stage highwaymen—as the idea of the long lonely mountain ride with the guide, that caused me to hesitate. The man was a full-blood Indian who was to escort me, and by the light of the lantern he looked very uncanny for a travelling companion with his sharp, little black eyes and hair, and striped blanket costume. However, I could not refuse when he handed me a note from Dr. R——, making a special request that I would take the case, as it was urgent, and required a trained nurse, both during the operation and after, and that he would accompany me from the foothills. Accordingly, giving my nursing "kit" into the arms of the Indian, whose name sounded like Mocca Julah, I climbed into the curious vehicle drawn by two mules.

### The Journey.

Arrived at the foothills we were met by Dr. R——, and my baggage transferred to the back of a mule and tightly strapped on with a "diamond cinch," which elicited a grunt from the poor beast. Dr. R—— then climbed on the back of another animal. He is a short, fat little man, and that is also the best description to give of the mule he bestrode. Then a tall and singularly angular beast of the same genus was led forward for my use. Horrors! no side-saddle. There was no help for it. Dr. R—— had started up the trail, and the Indian was growing impatient, so up I clambered, and at first attempted to keep on sideways; but when we came to a very narrow path overlooking a dizzy precipice I cast all squeamishness to the winds and bestrode the beast. How I wished myself safe at home! but there was no possibility to return, the path being too narrow. I often had to shut my eyes and resign myself to my fate at the worst places. As the day broke and the paths became wider I could not help enjoying the grand scenery. Magnificent trees, and huge granite boulders overgrown with all kinds of coloured lichens and wild flowers, past waterfalls whose music chimed in prettily with the bells on the mules. Occasionally the effect was changed from the "sublime to the ridiculous," when Dr. R——'s mule trumpeted forth a shrill bray, and standing stock still the little doctor would be seen clasping his arms round its neck to save himself from being shot over its head.

### The Patient.

At last we reached the hut where the wounded man was lying. A young medical student was there to administer the chloroform. We all set to work to make the place and necessary utensils as aseptic as possible under the adverse circumstances. A huge cauldron outside over a gipsy fire held plenty of boiling water. An Indian squaw stood ready to obey my orders, the first of which was to sterilise herself as far as possible by scrubbing her face, neck, arms, and hands. Going inside the hut I bathed my patient and prepared his crushed leg for amputation, placed everything to the best advantage, and, the instruments being sterilised, we donned our gowns. The doctors having neglected to bring theirs, I had to rig them and the squaw out in my white nightgowns—a proceeding that seemed to amuse the patient, a tall, swarthy, bearded Californian, who for natural hirsute covering would have rivalled Esau. Our make-shifts would have amused our English doctors. For sponges I had boiled some old towels and torn them up; for bandages, a sheet which had grown distinctly grey in its owner's service. Only one little wash basin was available, so we used two saucepans, and the frying-pan contained the instruments.

### The Indian Squaw Assistant.

At the first incision my Indian squaw assistant let forth a shrill war-whoop, which made us all jump; then she disappeared down the hillside. However, when the operation was over and the patient, whose name was "Sure-Shot Jack," was covered up, she returned and sat by him whilst I dug a grave for the amputated leg, which I could not get anyone else to touch. All the time I was there—a little over three weeks—she was a devoted helper, and, taking everything into consideration, moderately clean. She was a mother, and kept a very charming little pappoose hung up in a kind of flat basket against the wall. I don't believe it ever had a whole bath at one time, and I seldom saw it free from its swathing bands and in a state of nature. For the first week I was an object of awe and respect to the Indian population around, and I think to this hour they give the thermometer greater credit than the doctor for bringing the patient to an ultimate cure. One squaw daily brought a sickly boy, and offered me all sorts of curiosities to allow him to hold it "just a little bit."

### Nurse Left Alone.

The two doctors left soon after the operation, and gave me *carte blanche* in the matter of dressings. I must say I felt terribly lonely when they were gone. I do not suppose that Dr. R—— grudged the time it would have taken him to return to see the patient once more, but I am quite sure he did not want any more mule rides. His wife told me afterwards that he walked bowlegged for two days, and when sitting still had his legs tied together to straighten them. My patient was a rough diamond, and gave me as little trouble as possible. Nor had I any reason to complain of my treatment. My food was prepared by the squaw, and provided by her husband, the guide. He also did all the washing. My patient, when strong enough, regaled me with portions from his wild life, though he never divulged his real name. He had been lynched for cattle-stealing in Nevada, and cut down by an Indian girl. In fact, he had had many hair-breadth escapes. The walls and floor of his hut were covered with skins of animals he had killed—several wild cat and coyote skins, one buffalo hide, two mountain lions, two bears, and several deer skins.

### Rattlesnake in the Room.

My "beef tea" I made from venison as a rule; once from a haunch of bear brought in by some trappers. Quail and rabbits formed a great part of our meat diet. The Indian "Mocca" slept outside rolled in his blanket ready to assist me, and always carried out my orders in an ultra-solemn manner. I only once saw him smile, and that was when he came in, in response to a yell from me, and found me on the table, with my skirts closely gathered round me, while a large rattlesnake perambulated round the room. I have the rattle now, with its eighteen joints. He killed two more before I left, one in the back-room kitchen and one under the doorstep; also a tarantula and several scorpions.

### Recovery of the Patient.

My patient recovered, and when on leaving I presented my bill, pulled out a stocking from a hole in the floor and insisted on my accepting double the amount. He also impressed it on me that if ever I happened to be hard up I was to let him know, and he would take care I never wanted for long. Then, as I held out my hand to say good-bye, he raised it to his lips as respectfully and gently as any subject could to his sovereign. The Indian Mocca escorted me safely home, and when he left gave me a bracelet of wild cats' teeth and claws.

### The Treatment of Bed-sores in Different Stages.

### EXAMINATION QUESTIONS FOR NURSES—RESULT OF THE JANUARY COMPETITION.

### THE FIRST PRIZE.

"NURSE ISABEL" is again the successful candidate. She won the first prize in the November competition, and would have done so again in December, but she exceeded the permitted number of words. It will be well for all to read carefully her lucid explanation of what she considers the best general treatment for bed-sores. A marked characteristic of her answers is the manner in which she keeps to the matter in hand, instead of wandering off into branch railways of thought on side issues which have no important bearing on the question asked.

### THE SECOND PRIZE.

"Sussex" wins the second prize, though she would have done better to have given some alternative remedies, as different cases respond in totally opposite directions to the same treatment. Some drugs which have proved admriable in many instances will be almost poison to others. Moreover, it is well to change treatment for bed-sores frequently; red lotion for a few days, and then, if found suitable, copper lotion, returning again to red lotion. Sometimes great good may be effected by dropping all stimulating applications and trying simple castor oil dressing. It is a great mistake to carry conservatism so far as to continue one treatment, however good, *ad infinitum*.

### NURSE ISABEL'S ANSWER.

The progress of a bed-sore may be divided broadly into three stages: (1) The part affected presents a purplish-blue appearance, and the skin, not yet absolutely broken, is evidently on the verge of becoming so; (2) the margins of the sore will be observed to be breaking down from the still-living surrounding tissues, and sloughing commences and may continue till all the soft parts between skin and bone are destroyed; (3) sloughing over and the sore clean. Granulation proceeds until healing is completed. Certain general principles must now be laid down, and remembered throughout. Pressure on the part must in every devisable way be avoided. The vitality of surrounding tissue, up to the very edges of the sore, must be maintained by gentle massage. All dressings, other than dry ones, must be cut the shape and size of the sore, so as not to overlap on to sound tissue. They must be so applied that they remain where they are placed, and are not found promiscuously in the bed. When the bed pan is given, a pad of wool covered with jaconet must be placed between the edge of the pan and the patient's back, if the sore is in this region. The absolute necessity of cleanliness and dryness need not again be insisted on. Treatment for stage 1—As long as the skin is unbroken, protect part with thin pad of cotton wool, covered with soft linen, and keep in place with two or three strips of ribbon strapping, placed crosswise. The strapping should be an inch longer either way than the dressing. 2—Apply fomentations of boracic lotion, or thin linseed poultices, spread on linen; cover with jaconet. Use bandage, binder, or strapping for keeping it in place, as position and size of sore, &c., determine. Four thicknesses of lint must be used for fomentation, and renewed as often as condition of patient allows. Poultices should be changed every four, preferably every two, hours. Syringing with antiseptic lotion—boracic, Condy or Jeyes' are good in the district—and occasional powdering of sore with charcoal or iodoform is useful before poulticing. 3—Stimulation is the keynote, and the granulations appreciate variety of treatment. Resin ointment spread on lint, carbolic, benzoin, or iodine lotions, and antiseptic gauze for filling the sore, may all be used. If the granulations are pale, flabby, or too high, they may be touched with a nitrate of silver pencil. If perfectly healthy, grafting may sometimes assist the final state of cicatrisation. The progress made corresponds, as a rule, with the patient's general condition. If the age and illness of patient render the sore hopeless when first seen, it will be unkind to attempt more than the relief of pressure, and the application of some simple antiseptic dressing. The question of pressure is generally one which taxes the nurse's ingenuity to the utmost; but by pillows, air cushions, and pads, which she will make herself to meet the special requirements of the case, the difficulties may be overcome.

### "SUSSEX'S" ANSWER.

When a bed-sore is once formed, *i.e.*, when the skin is once broken, there are three things which must be borne in mind, and to which unflagging attention must be given. First, stimulation of the surrounding parts; secondly, the patient's general health; thirdly, the avoidance of friction. The latter must be managed by means of the patient's position, or by ring pads or some similar contrivance. It should be remembered that stimulation of the circulation in the parts liable to bed-sores is quite as important when a bed-sore has actually formed as it was before the skin was broken. Indeed, it is even more so. Therefore soap and water and spirit must still be ungrudgingly used. The latter, of course, must not be allowed to touch the spot where the skin is broken, but the soap and water should be used, with care, over the whole surface. There are various remedies which may be tried for the healing of the sores themselves. For a simple, superficial bed-sore nothing is better than ordinary zinc ointment, gently rubbed in. For deeper or longer-standing ones, lint soaked in red lotion and cut the exact size of the sore, covered with protective, and changed two or three times a day, gives very good results. Old or very dirty bed-sores should be first poulticed or fomented with hot water, weak carbolic (cautiously used), or weak perchloride of mercury or chlorinate of soda.

### A NEW RULE TO COME INTO PRACTICE FOR THE FEBRUARY QUESTIONS.

The number of answers sent in have decided the examiners to give honourable mention cards to those who have sent in exceptionally good papers, but have failed to secure either of the two prizes. These will only be used occasionally when the answers reach a high standard. In the case of one candidate winning the first prize three times in the same year she must after that success understand that her prize will pass to the second best candidate, though her paper will be published. Thus, Nurse Isabel has been successful twice in three months, and should she again come out first before May she must rest on her laurels for a time after that date. It is, however, hoped that such successful candidates will continue to compete, because their papers are useful to others.

Last month a very good paper was sent in by a male nurse, who narrowly escaped securing a prize. It is very desirable that male nurses, who are such useful members of the profession, should enter into the competition.

### QUESTION FOR FEBRUARY.

Define the different degrees of danger to life from various kinds of severe burns and scalds, and the means you would take to obviate fatal results. N.B.—You are not asked how you would treat the burns themselves.

### Echoes from the Outside World.

### AN OPEN LETTER TO A HOSPITAL NURSE.

Lord Roberts and Lord Kitchener have gone to the ront, but the position, so far as the war is concerned, continues one of suspense, and the dearth of intelligence is emphasised by the prominence given to an interchange of etters between Messrs. Kruger and Steyn and Lord Roberts, in which the British Field-Marshal scores heavily, and completely turns the tables upon the two Presidents. Instead of admitting that our troops have been guilty of the devastation and pillage of Boer homesteads, Lord Roberts accuses the Boer forces of offences which can easily be reproved, and admonishes the Presidents to see that they do not repeat them. In the absence of news from South Africa, the interest lies centred in the remarkable result of the York election and the division in the House of Commons on Tuesday night. Unhappily, the Leaders of the Opposition would not, or could not, act on the advice of a large section of their supporters, who wished the amendment withdrawn, but the mischief caused by want of union in Parliament is minimised by the immense majority against it. More striking, however, as everybody seems to think, than the rejection of the hostile amendment by 213 votes, is the return of Mr. Faber for York by a majority of 1,430. For once we may assume that a single constituency is speaking the voice of the nation, since Lord Charles Beresford, most popular of candidates, could only obtain a majority of eight. In other words, at least a thousand electors voted Unionist because they approve the policy of the Government in respect to the war. Even Presidents Kruger and Steyn can hardly be blind to the significance of this incident.

The financial difficulties of the Boers in carrying on the war, which some people think are about as serious as any they have to encounter, are illustrated by the appeal issued to the "world" for funds to save "the little nation" from famine, by the heavy fines which are levied on those who wish to escape being commandeered, and by the imposition of a special war tax on non-residents, companies, and syndicates, and on persons acting as trustees. It has always been urged by financial authorities who are particularly well acquainted with the resources of the Transvaal Government that time is on our side; and, though I am sure that none of us, not even nurses who are gaining honour and glory by their devotion to the sick and wounded soldiers, wish that the campaign should be prolonged a single day beyond the period where peace is practicable, it is some consolation to know that the generosity and the public spirit of the country will prevent any cause for anxiety about the success of war so far as the British are concerned. On the question of time, a significant statement has been made by the High Commissioner, Sir Alfred Milner, who was asked by the Ex-Lord Mayor of Belfast whether he desired that a further appeal should be made to the citizens for money, replied in the negative, and said that "with strict economy," he has sufficient for another four or five months, "by which time he trusts war will be over." As he is in a position to judge, and would, of course, weigh his words, it may be assumed that he too recognises that, apart from the victories which we are waiting for, there is the chance of the Boers being crippled by the absence of both cash and credit.

Even those of us who do not care much about reading reports of speeches in Parliament have been struck with the fact that the debate on the Vote of Censure has been the means of bringing at least one man to the front. And a very interesting personality he is, too. I refer, of course, to Mr. George Wyndham, about whose brilliant success everyone has been talking for the last few days. You know that Mr. Wyndham is Under Secretary for War, and is supposed by some to be largely responsible for the nursing arrangements of the Army. As a matter of fact, he has to leave such things to the Army Medical Department, and concern himself with more general issues. However, though the Under Secretary neither interviews, nor is interviewed by, nurses, you may care to learn that he is a singularly attractive-looking man, with the most delightful manner and a voice to match. These are advantages that count more in the drawing-room than at Westminster, but Mr. Wyndham has shown that the charms with which nature has endowed him are supplemented by remarkable clearness of vision, discreet candour, consummate tact, and very considerable oratorical powers. As he is still on the sunny side of forty, he ought to have a big future before him; and, other things being equal, it will not tell against him that he is the grandson of a peer, the husband of the Countess Grosvenor, the stepfather of the young Duke of Westminster, and possesses an ample private fortune. Like many of our most illustrious men, he began his career as a soldier, and served his apprenticeship to politics as private secretary to a Cabinet Minister. He himself is now on the threshold of the Cabinet.

There can be few women who are not interested in the coming public celebration of the pioneer public school for girls. Probably some of you were educated at the Frances Mary Buss Schools, which were founded fifty years ago, on April 4th. The event will be commemorated by a service of thanksgiving at St. Paul's Cathedral on the evening of the 3rd, with a sermon by the Archbishop of Canterbury, to which, I am told by Dr. Sophie Bryant, the head mistress of the North London Collegiate School, that invitations have been issued to more than 3,000 old pupils, while the pupils and teachers at the Upper and Lower Schools will exceed 1,000. As a great many parents and friends also wish to be present, even the vast resources of St. Paul's threaten to be severely taxed on the occasion. Then, on the Day of Jubilee, there will be receptions at both schools morning, afternoon, and evening, winding up with a gymnasium display. Mrs. Bryant, you will learn with interest, believes that the remarkable development of the movement, which was started in quite a humble manner by the late Miss Buss, has largely been due to the use of public examinations. The very first year that the Cambridge examination was open to girls Miss Buss sent up twenty-five girls to the University. To her unwearied labours our sex are much indebted for the strides which have been made in respect to the higher education of girls, and it is a matter for congratulation that her able successor has been well content to pursue the same policy, while never losing sight of the requirements of the day.

The wisdom of Mr. Alexander in eliminating the lying-in-state scene which, on the night of its production, terminated "Rupert of Hentzau," is obvious. It was a spectacle which should not have been introduced on the stage, but by recognising the mistake directly it was pointed out the manager of the St. James's Theatre has made the best amends he could. The piece is now not only absolutely unobjectionable, but I can strongly advise a visit to the house. Many of you have probably read Mr. Anthony Hope's sequel to "The Princess of Zenda," as well as seen the dramatic version of the latter; and in some respects "Rupert of Hentzau" is more interesting as a play than the earlier work. The duel in the cellar between Rassendyll and Hentzau is the most exciting of several clever scenes, but it is the bright and incisive dialogue which constitutes its chief attraction. The acting also, especially that of Mr. Alexander as Rassendyll, Mr. Vernon as Sapt—the central figure of the play—and Miss Fay Davis as Queen Flavia, is admirable. The gowns worn by Miss Davis are quite lovely.

### The Nurses of the Imperial Yeomanry Hospital.

The full list of the nurses appointed to the Imperial Yeomanry Hospital will be found below. It will be seen that pains have been taken by the Committee of Selection to secure a representation of the provincial hospitals. We are able, in fact, to state on authority that the utmost care has in all respects been exercised in order to obtain for the sick and wounded soldiers in South Africa the services of the most capable, the most experienced, and the most suitable in every sense, of the nurses available. This does not imply that the supply of capable, experienced, and suitable is limited, for if 40 more were wanted to-morrow they could be secured at once. But it does mean that out of the more than 700 nurses who volunteered for the work every effort has been made to test their fitness in all points, and that while social influence was entirely ignored, training and capacity were primarily considered. The ladies of title connected with the hospital did not, it was only fair to say, attempt to put the slightest pressure on those who were charged with the task of weighing the qualifications of the applicants. Even in the case of enthusiasts who came forward with offers to provide a nurse and pay for her support, the nurse had to stand in with the others, and in many instances it was found impossible to engage her, though in none did the nominee, as a consequence, withdraw her offer to contribute. A number of generous ladies are, therefore, still able to say that they are supporting a particular nurse, though she was chosen by the committee. A most pleasing feature has been the enthusiasm manifested by the whole of the nurses. There was not one applicant who was not keen to go to South Africa nor unprepared to submit to the undoubted hardships which have to be faced.

At the same time the committee justly pride themselves on the fact that instead of nine nurses for 520 beds they have thirty-nine. As a rule, the sisters in the base hospitals can do little actual nursing, but with regard to the Imperial Yeomanry Hospital it is obvious that this will not be the case. If, for instance, out of the 520 patients there are 200 practically convalescents, two nurses will fairly well suffice to look after them, leaving a good margin for the remainder. There will, of course, be a number of orderlies, but it is pleasant to observe that the principle of increasing the proportion of female nurses in the military hospitals is here distinctly recognised. Although no matron is actually on the committee, one of the best known hospital matrons in London has throughout acted in an advisory capacity. Miss Fisher, the matron superintendent, and Miss Brereton, the night superintendent, are the heads of the nursing staff, but the duties of the matron superintendent will not be of quite the same character as those of an ordinary hospital matron. The nurses will be attended upon by ten servants, classed as ward-maids, and a housekeeper—Miss Winifred Cheesman—will accompany them. They are now members of the Army Nursing Reserve, and have had at least three years' training, though before their selection only a few of them belonged to the service. Their remuneration is £40 a-year and £20 bonus, with free passage, maintenance, lodging, and £8 for the necessary equipment. The engagement is for a year, and in some instances the places which they vacate will be kept open for them till they return. They all start in the "Guelph" on the 17th, or a week later than the medical officers. The following are names of the nurses, with some details of their experiences:—

Miss Mary Colborne Fisher (the matron superintendent) was trained at Guy's Hospital, where she was subsequently Sister "Lazarus" for nearly five years. She has since been matron of the Cottage Hospital at Watford, and superintendent of the Convalescent Home at Saltburn-on-Sea. She holds the Guy's Medal.

Miss Katherine Blanche Brereton (night superintendent). Miss Brereton was trained at Guy's Hospital, was staff nurse at Birkenhead Children's Hospital, holds the midwifery certificate of the General Lying-in Hospital and the certificate of the L.O.S., and has since been sister of "Bright" ward at Guy's for some years.

Miss Sarah Barnes was trained at Guy's Hospital, has been staff nurse since November, 1895, and holds the Guy's medal.

Miss Edith Beedie was trained at the Victoria Hospital, Burnley, and has since been engaged in private nursing.

Miss Seabrook Cooper has been three years at the Royal Infirmary, Liverpool, and three at the East Suffolk Hospital.

Miss Edith Cheetham was trained and has been at Guy's Hospital for four years.

Miss Adeline Cable was trained at the London Hospital, with which she has been connected for eight years. She is now at the Nursing Home.

Miss Florence Coates was trained at the London Hospital.

Miss Gertrude Fletcher was trained, and has been six years at, Prince Alfred's Hospital, Sydney.

Miss Charlotte Gash was trained at the Warneford Hospital, Leamington.

Miss Agatha A. C. Higgs was trained at the North London Hospital for three years, and was subsequently charge nurse for two years. She entered the Royal Hospital, Waterloo Bridge Road, as staff out-patient nurse in November, 1897.

Miss Ethel Harmer was trained at Guy's Hospital, and has since been one of the private nurses attached to Guy's Institute.

Miss Jane Hodge was trained at St. Bartholomew's Hospital.

Miss Alice Hislop has been three years at the Royal Infirmary, Dundee, and three years at the Children's Hospital, Edinburgh.

Miss Gertrude Johnston was trained at the Royal Infirmary, Liverpool.

Miss Ethel Keene was trained at St. Bartholomew's Hospital, and has been nursing at Shadwell for two years.

Miss Barrie Lambert was trained at the London Hospital.

Miss Bertha Lancaster was trained at University College Hospital.

Miss Edith Lee was trained at the London Hospital.

Miss Evelyn Leggatt was trained at King's College Hospital, where she is now sister.

Miss Mary Louisa Loveday was trained at Guy's Hospital, and has been sister since November, 1898.

Miss Elizabeth Macpherson was trained at Cumberland Infirmary, and has had fever nursing experience in the London North-Eastern Hospital.

Miss Lilian Mustard was trained at the Northampton General Hospital, and has been nursing in Egypt for a year.

Miss Mary O'Neil was trained and has been at Guy's Hospital three years and eight months.

Miss Ada Pierce was trained and has been at the Middlesex Hospital four and a-half years.

Miss Margaret Penrose was trained at Sussex County Hospital, Brighton, where she was subsequently staff nurse. From May, 1889, to April, 1891, she was at St. Bartholomew's Hospital. Her other experience has included that of matron for a year of Harlington Cottage Hospital, and since December, 1894, she has been engaged in private nursing in connexion with Princess Christian's Nurses' Home at Windsor.

Miss Dora Pryde was trained at Kensington Infirmary, and has had four months' experience in the Fever Hospital.

Miss Mary Powell was trained at Guy's Hospital.

Miss Rosamond Grace Rolleston was trained at the Hospital for Sick Children, Great Ormond Street, the National Hospital for the Paralysed, Queen Square, and St. Bartholomew's Hospital. She was staff nurse at "Barts" from May, 1892, to February, 1893, and has been sister of Elizabeth ward since that date. Miss Rolleston won the Clothworkers' Company Book Prize in 1890.

Miss Minnie Ellis Rowell was trained at Guy's Hospital, and has since been one of the private nurses attached to Guy's Institute.

MISS HILDA ROOKE was trained at Radcliffe Infirmary, Oxford, and has been nurse at St. George's Hospital for a month.

MISS MARY DOUGLAS STEPHENSON was trained at Charing Cross Hospital, where she was subsequently sister for five years. She has since been matron of Tiverton Infirmary and Chalmer's Hospital, Edinburgh.

MISS GRACE TILLOTT was trained at Guy's Hospital, and has since, for two and a-half years, been one of the private nurses attached to Guy's Institute.

MISS ANNIE TIMBRELL was trained at Guy's Hospital, and has since, for three years, been one of the private nurses attached to Guy's Institute.

MISS ELEANOR TUCKER was trained at Leicester Hospital, and has had two years' experience in fever nursing.

MISS ELIZA UPPLEBY was trained and has been at the Middlesex Hospital four years.

MISS ELLEN VINCENT was trained at Guy's Hospital.

MISS ELEANOR WALKER was trained, and has been, at Edinburgh Royal Infirmary for four years and a-half.

MISS ANNIE WALKINSHAW was trained at St. Bartholomew's Hospital, and has since been three years and a-half at Kensington Infirmary.

### Nursing in Naval Hospitals.

THE following is the report presented at the last meeting of the Parliamentary Bills Committee of the British Medical Association from Inspector-General Turnbull on the nursing in naval hospitals :—

The nursing in naval hospitals is performed at Haslar, Plymouth, Chatham, Malta, and in the sick quarters for naval cadets at Dartmouth, by sisters of the nursing staff and by male nurses of the sick berth staff.

In the other naval hospitals, at home and abroad, the male nurses specified are alone provided.

The sick berth staff is recruited from the shore, the age for entry being from twenty-one to twenty five years. The recruits must possess a fair knowledge of reading, writing, and the simple rules of arithmetic, and be considered physically fit; but at present there is no fixed standard for height or chest measurement.

On entry they undergo at Haslar a course of instruction in nursing by the nursing sisters and senior sick berth staff; in "first aid" and ambulance drill by a naval surgeon; in compounding by the senior dispenser; in cookery for the sick by a lady cook; in physical drill by an instructor.

When qualified to pass an examination in these subjects, they are confirmed in the rating of sick berth attendants, and advance by seniority, efficiency, and good conduct to sick berth steward and to chief sick berth steward rank, namely, that of a chief petty officer in the Navy.

They are eligible for pensions after twenty-two years' service, and may, if considered desirable, serve to the age of fifty, indicating continuous service as contrasted with short service in the army.

As regards messing, all naval ratings have rations, or an equivalent money payment in lieu; provision is made for the usual three meals a day; there are no special arrangements for refreshments when on night duty.

Night duty when required is, as a rule, in watches of three hours, from nine p.m. to six a.m., and in addition to day duty.

Such are some of the present regulations for nursing in naval hospitals, but the subject is now under special consideration by the Admiralty, and it is understood that great reform and improvement, both in the case of the nursing sisters and the sick berth staff regulations, have been recommended, and may shortly be carried out with a view to make these services more attractive than at present, the necessity for which being recognised by the Admiralty.

ALEX. TURNBULL,
Inspector-General of Hospitals and Fleets (retired).

### The Princess of Wales's War Fund.

WE publish to-day the final list of subscriptions :—

Amount previously received, £225 5s. 4d.

| | s. | d. |
|---|---|---|
| Nurse Davies, P.F. ... | 5 | 0 |
| Nurse A. S. Passmore, P.F. ... ... ... | 1 | 0 |
| E. K. W. ... ... | 5 | 0 |
| M. M. S. ... ... | 2 | 6 |
| Nurse Gosling ... ... | 2 | 6 |
| D. M. D. O. ... ... | 1 | 0 |
| B. Thomson, P.F. ... | 1 | 0 |
| M. Nye ... ... ... | 5 | 0 |
| Sister Hayes ... ... | 5 | 0 |
| Nurse M. Weir, P.F.... | 2 | 6 |
| Nurse Ratcliffe ... | 1 | 0 |
| L. Young, P.F. ... | 5 | 0 |
| A. Young, P.F. ... | 5 | 0 |
| M. M. Aspinall ... | 3 | 0 |
| Collected at the Mile End Infirmary, Bancroft Road, N.E. :— | | |
| Sister Hartwell ... | 1 | 0 |
| Sister Craddock ... | 2 | 0 |
| Sister Brown ... | 1 | 0 |
| Sister Baxter ... | 1 | 0 |
| Sister Craig ... ... | 1 | 0 |
| Sister Berry... ... | 1 | 0 |
| Sister Dingle ... | 1 | 0 |
| Sister Grange ... | 1 | 0 |
| Nurse Reed ... ... | 1 | 0 |
| Nurse Shearer ... | 1 | 0 |
| Nurse Boyce ... | 1 | 0 |
| Nurse Purdge ... | 1 | 0 |
| Nurse Blanche ... | 1 | 0 |
| Nurse Humphrey ... | 1 | 0 |

| | s. | d. |
|---|---|---|
| Nurse Jones... ... | 1 | 0 |
| Nurse Harding ... | 1 | 0 |
| Nurse Townsend ... | 1 | 0 |
| Nurse West ... ... | 1 | 0 |
| Nurse Maughan ... | 1 | 0 |
| Nurse Rudd ... | 1 | 0 |
| Nurse Howard ... | 1 | 0 |
| Nurse Morphey ... | 1 | 0 |
| Nurse Chester ... | 1 | 0 |
| Nurse Donnovan ... | 1 | 0 |
| Nurse Richards ... | 1 | 0 |
| Nurse Lensey ... | 1 | 0 |
| Nurse Brindley ... | 2 | 6 |
| Nurse Dudley ... | 1 | 0 |
| Nurse Stent... ... | 1 | 0 |
| Nurse Lyth ... ... | 1 | 0 |
| Nurse Perry ... | 1 | 0 |
| Nurse Forsyth ... | 1 | 0 |
| Nurse Turton ... | 1 | 0 |
| Nurse Hayes ... | 1 | 0 |
| Nurse Evans ... | 1 | 0 |
| Nurse Robb ... ... | 1 | 0 |
| Nurse Gall ... ... | 1 | 0 |
| Nurse Potier ... | 1 | 0 |
| Nurse King ... ... | 1 | 0 |
| G. Nye ... ... | 2 | 0 |
| A. H. R. ... ... | 2 | 0 |
| J. Todd ... ... | 1 | 0 |
| F. W. B. ... ... | 2 | 6 |
| Policy 3,743 .. | 5 | 0 |
| L. H. B. ... ... | 2 | 0 |
| Nemo ... ... | 2 | 0 |

Total, £230 7s. 10d.

### Minor Appointments.

ST. ANNE'S HOME, HERNE BAY.—Miss Mabel Watson has been appointed Charge Nurse. She was trained at Chelsea Infirmary for three years.

ROYAL VICTORIA EYE AND EAR HOSPITAL, DUBLIN.—Miss Anna Sproule has been appointed Staff Nurse. She was trained at Sir Patrick Dun's Hospital, Dublin.

HOLBORN UNION INFIRMARY.—Miss Annie Bates has been appointed Nurse. She was trained by the Meath Workhouse Attendants' Association at St. Peter's Home, Mortimer Road, Kilburn.

FULHAM INFIRMARY NURSES' HOME.—Miss Tabitha Alfreda Bodington has been appointed Home Sister. She was trained at Charing Cross Hospital and the Children's Hospital, Nottingham.

BLACKBURN FEVER HOSPITAL.—Miss Ina Oldfield has been appointed Charge Nurse. She was trained at Calverley Moor Hospital, Bradford, and has since been nurse at the Borough Hospital, Halifax.

ST. ALBANS UNION INFIRMARY.—Miss Maud Culyer has been appointed Assistant Nurse. She was trained by the Meath Workhouse Attendants' Association at the Home for Invalids, Aubert Park, Highbury.

DORSET COUNTY HOSPITAL, DORCHESTER.—Miss Margaret Hope Stewart has been appointed Charge Nurse. She was trained at Cumberland Infirmary for three years, medical and surgical, and has since been staff nurse in a surgical home in London.

POPLAR AND STEPNEY SICK ASYLUM.—Miss Sarah Kilgallen has been appointed Night Sister. She was trained at the General Infirmary, Sheffield, has since been engaged in private nursing, and for eighteen months has been charge nurse at the Fountain Fever Hospital, Tooting.

### Everybody's Opinion.

[Correspondence on all subjects is invited, but we cannot in any way be responsible for the opinions expressed by our correspondents. No communication can be entertained if the name and address of the correspondent is not given, as a guarantee of good faith but not necessarily for publication, or unless one side of the paper only is written on.]

### EASY FRAUDS.

"The Matron of a Northern Infirmary" writes: The easy frauds of which your correspondents complain must have been going on for some time. The same person (I judge from the description) called on me in 1896 (presumably sent by a lady friend living in the town) to inquire about a nurse for her mother. She had called at the nursing home, she said, and there was not a nurse available. She told me much the same as she had told others, and after a time she asked for her train fare, as her daughter had gone home by an early train and taken the bag which contained her purse. She had various other requirements all urgently needing to be met. Her knowledge of people in the town and matrons of hospitals was large and correct. She left me two addresses, one at Ambleside, the other at Ladbroke Grove, Notting Hill. Of course, both letters were returned through the usual channel.

### CHRONICS AND CONVALESCENTS.

"Hors de Combat" writes: In answer to "A Convalescent Nurse," may I be allowed to say how pleased I was to see a subject brought forward which should so entirely enlist our sympathies? Those who are blest with health and strength can scarcely realise the utter weariness of body and mind when the body lies helpless and passive day by day with no definite hope of recovery. Nor to what depth of dreariness the spirit sinks for want of a few kindly and apt words of compassion which cheer and brace the soul. Also, taking advantage of this opportunity, may I be allowed to plead most earnestly for gentle and courteous treatment of repugnant or loathsome "cases"? Do not let us hurry over the duties which the nature of their disease entails upon us, turning, with thinly-veiled relief, to a more congenial task. Let natural instinct and moral pride be subjugated to cultured compassion, then we shall be a little more like the loving Christ, who dared to touch the unclean lepers.

### THE MONOTONY OF PRIVATE NURSING.

"Sympathy" writes: "An Admirer of Florence Nightingale" evidently craves for excitement, and lacks the true womanly sympathy and patience which are so requisite for the noble profession of nursing. She probably imagines the daily "uninteresting duties" connected with all nursing would be less "monotonous" if the patients were "wounded and dying soldiers" than nervous old ladies! Possibly Florence Nightingale's "Admirer" has had more experience with "les malades imaginaires" than with more serious cases? As a nurse of thirteen years' wide experience under some of our most distinguished London doctors I can truthfully assert that I never found private nursing monotonous, nor have I come across any nurse who found her "duties uninteresting" if her heart were in her work. Unfortunately too many nurses take up this noble work for excitement, greater facility for familiar intercourse with the opposite sex, or weariness of home duties, which, to their small natures, would also doubtless seem "uninteresting." Possibly a little personal experience of the monotony of a long illness might make the writer more sympathetic with even the "fads and fancies" of a nervous patient.

### ST. GEORGE'S INFIRMARY, FULHAM ROAD.

"A Lady Visitor" writes: I desire, as a visitor at St. George's Infirmary, to say that I quite sympathise with the desire to improve the present condition of the patients, and I think that reforms in the general management are urgently needed.

"Annoyed" writes: May I beg for a small space in your paper to refute some of the accusations against the management of the St. George's Infirmary? Having been a nurse there, I can say that I have never heard any of the patients complain of insufficient food, and all appeared satisfied. Considering it is a Poor Law infirmary, and not a well-supported hospital, the patients fare very well. As regards the nurses' food, in my opinion it is excellent, plentiful, and a good variety. The last meal is not at four o'clock, as stated. Supper is served from half-past seven to eight o'clock, and if a nurse is out on a day's leave her supper is kept until her return at ten p.m., so the nurse does not go to bed hungry as stated. I have never known nurses to wear damp clothing, I give them credit for better sense. The nursing staff is small owing to lack of accommodation. There is a nurses' home being erected, and when finished it will be equal to any in London. I have never known "artizans' children" go into the workhouse infirmary, unless the lady visitor means "pavement artists'" children. Some of the children's morals on entering the infirmary are not all that can be desired, but before they leave it they are refined and well-mannered, owing to the nurses' tuition and kindness, such as some of them have not known before. It would be much kinder if the lady visitors would complain to those in authority before making a public talk of the place for the sake of a name.

### DANCES IN HOSPITALS.

"A Loyal Nurse" writes: The question of dances in hospitals is being so warmly discussed by matrons at the present time that a view of the subject from a nurse's standpoint will scarcely be amiss. My standpoint is that of a nurse, a third-year probationer, in what I know to be one of the largest, and believe to be the best managed of London infirmaries. Our matron and our medical superintendent are both very strict disciplinarians—at times a little too strict for immediate appreciation—though in the main we all feel the better of it. They are well supported by their assistants. We—the staff—consist of the usual mixture of girls and women to be found in such institutions, who have taken up nursing, not from philanthropic motives, but as a doctor takes up his profession or a lawyer his—as a means of earning our bread. Because it so happens that in our work our womanliness and natural gentle qualities are in constant requisition, it surely does not follow that we do not need ordinary relaxation, and occasionally a little extra-ordinary pleasure, such as the much-matron-condemned annual dance? I think that without being guilty of self praise I may say we deserve it. We work very hard; our duty hours are long; we spend six months out of twelve on night duty, which, to say the least of it, is very depressing; we continually have to do extra work, in cases of a fellow nurse being temporarily off duty, &c. But, here at all events, we have never shown ourselves unwilling to meet any extra demand made on our energy, or even on our time, and how precious a nurse's off-duty time is no one but a nurse can tell. This is our work. Our play is three weeks' annual leave. I do not wish in any way to dictate a course of action to matrons. As a new probationer I had an inclination that way, but I know better now! But I fail to see how a well-organised dance among otherwise well-disciplined nurses could give rise to the unpleasanteries of which your matron correspondents of last week complain. Of course, there are dances and dances. There are those to which the doctors only invite their friends. People who expect such dances to be successful cannot be very far-seeing. To begin with, no self-respecting nurse would go to supply the doctors' gay bachelor friends with a night's amusement. But a dance, to which the nurses as well as the doctors invite each a friend—subject, of course, to the matron's approval—and from which patronage and condescension are abolished, and all meet on a thoroughly friendly, social footing, could not and does not do harm, or in any way interfere with our interest in our work, or our respect for proper authority. On the contrary, we return to our work with renewed energy, we feel more friendly towards each other, and more attached to our training school and such feelings are not, unless I am much mistaken, a groundwork for "disloyalty."

### For Reading to the Sick.

"The garment of praise for the spirit of heaviness."—*Isa.* lxi. 3.

"As sorrowful, yet always rejoicing."—2 *Cor.* vi. 10.

Hope on, hope on, the golden days

Are not as yet adawning,

The mists of night

Precede the light,

And usher in the morning.

Hope on, hope on, through frost and snow,
Through trouble, toil, and sorrow;
Through wind and rain,
And tears and pain,
The sun shall pierce to-morrow.

Hope on, hope on, though friends be few,
And dark the way before thee.
A God of love
From heaven above
Shall shed His radiance o'er thee.

—*Godfrey Thring.*

**Reading.**

He who possesses the grace of cheerfulness prays better than those who are sad, and is more readily united to the Holy Spirit of God, which is the spirit of joy; because like ever draws to like.—*Bonaventura.*

There is no moral imbecility so great as querulousness and sentimentality.

Joy is the lifelong morning of our souls, an habitual sunrise out of which worship and heroic virtue come.

All joy lies in union with God; all sorrow in exile from Him.

Joy clings to us as the creatures of God; it adheres to us wherever we go; its sunshine lights upon us and gives us an attractiveness above that which is our own. . . . Without speaking of God it teaches Him; it leaves tranquillity behind, and not unfrequently sweet tears of prayer. All things grow silently Christian under its reign. It brightens, softens, transfigures, like the sunlight, the most improbable things which come within its sphere.

Those bright souls to whom the gift of cheerfulness is given, have a great work to do for God, and they do it often when they realise it least; they have a light within them which was not delusive when they were young, and which age will only make more golden without eliminating its heat. Truly, he is the happiest, the greatest, and the most Godlike of them, as well as the sole poet among men, who has added one true joy to the world's stock of happiness.—*Faber.*

Cheerfulness is marvellously infectious. A bright happy soul rejoicing in all God's gifts, seeing cause for thankfulness and gladness in everything, counting up mercies rather than trials, looking at the bright side, even of sickness, bereavement, and death. What a very fountain of goodness and love of Christ such a one is.

I remember one who, worn with sickness and sleepless nights, answered to the question if the night did not seem interminable, "Oh, no. I lie still and count up my blessings." That was surely worth many sermons on thankfulness. . . . Holy joy has indeed a very communicative property, and no wonder, inasmuch as it is a peace which comes because those in whom we find it are "stayed on God."—*H. S. Lear.*

Enjoy the blessings of this day, if God should send them; and the evils of it bear patiently and sweetly; for this day only is ours. We are dead to yesterday, and we are not yet born to the morrow.—*Jeremy Taylor.*

### Notes and Queries.

The contents of the Editor's Letter-box have now reached such unwieldy proportions that it has become necessary to establish a hard and fast rule regarding Answers to Correspondents. In future, all questions requiring replies will continue to be answered in this column without any fee. If an answer is required by letter, a fee of half-a-crown must be enclosed with the note containing the enquiry. We are always pleased to help our numerous correspondents to the fullest extent, and we can trust them to sympathise in the overwhelming amount of writing which makes the new rules a necessity.

Every communication must be accompanied by the writer's name and address, otherwise it will receive no attention.

*Military Training.*

(188) "Inquirer" is anxious for military training. Will you tell her at what military hospital probationers are taken, and also the terms?

No probationers are trained at military hospitals. Fully-trained nurses have a supplementary course at Netley after joining the Army Nursing Service.

*Calcutta.*

(189) Can you give me any further particulars or information respecting the "scarcity of nurses in Calcutta," as I should like to apply for a post if they are increasing the staff?—*R. E. Harris.*

We have not heard of any scarcity of nurses in Calcutta. If you desire work in India, why not write to the secretary of the Colonial Nursing Association, Imperial Institute, S.W., and to the hon. secretary, The Up-Country Nursing Association, The Cedars, Strawberry Hill.

*Epileptic.*

(190) Will you kindly give me the address of institutions suitable for an epileptic working woman?—*G. M.*

The National Society for the Employment of Epileptics, 12, Buckingham Street, Strand, W.C. Home for Epileptics, Maghull, near Liverpool. The National Association for Promoting the Welfare of the Feeble Minded, 53, Victoria Street, S.W.

*Books.*

(191) I want to get a good book on nursing. Would you tell me if "Nursing," by Isabel Hampton, is thorough, clear, and fairly comprehensive?—*M. E. G.*

Isabel Hampton's is a standard work on nursing, and a great favourite. It is published by the Scientific Press at 7s. 6d.

*Midwife.*

(192) I am a trained midwife, and would be glad if any nurse would tell me where I would get work in country town or village.—*Nurse Mary.*

See our advertisement columns.

*Blind.*

(193) A widow with extremely limited means would be glad of any information as to where she could place her son, aged 17, with a view to having him taught a trade. He is quite deaf, and is going blind.—*E. M. Littlewood.*

Gardner's Trust for the Blind, 53, Victoria Street, Westminster, seems the most likely of many charities to help. See Burdett's "Hospitals and Charities" (5s. Scientific Press) for full list. A copy can be seen at the Office.

*Chip Carving.*

(194) Could you inform me where I could get lessons in chip carving, and what the cost of same would be?—*Private Nurse.*

You would possibly be able to obtain instruction at any of the polytechnic colleges at a trifling cost. There is one in Regent Street.

*Paralysed.*

(195) Would you kindly let me know of any homes where a paralysed man could be received, preferably without payment, and where he would be allowed to smoke, which is his only enjoyment?—*E. F. B.*

There are very few homes that receive incurable patients without payment, and those that do generally elect their inmates by subscribers' votes. The National Hospital for the Paralysed and Epileptic, Queen's Square, Bloomsbury, W.C., grant pensions to some deserving cases; or such a case would be eligible for election at one of the homes for incurables.

*Army Nursing Reserve.*

(196) Will you kindly tell me how a fully-trained nurse can become a member of the Army Nursing Reserve?—*H. N. R.*

Apply to the hon. secretary, Army Nursing Reserve, 18, Victoria Street, Westminster.

*Maternity Training.*

(197) Where can I obtain a short training in maternity with a view to qualifying myself for district work? I cannot afford to pay fees, nor to wait long.—*Widow.*

There is no free training in maternity work. Either the candidate pays fees, as in the public institutions, or gives her services by arrangement for a longer or shorter period to a private one. See advertisements for associations offering to pay fees in return for district services afterwards.

*Standard Books of Reference.*

"The Nursing Profession: How and Where to Train." 2s. net.

"The Nurses' Dictionary of Medical Terms." 2s. 6d. net.

"Burdett's Series of Nursing Text-Books." 1s. each.

"A Handbook for Nurses." (Illustrated.) 5s.

"Nursing: Its Theory and Practice." New Edition. 3s. 6d.

"Helps in Sickness and to Health." Fifteenth Thousand. 5s.

All these are published by The Scientific Press, Ltd., and may be obtained through any bookseller or direct from the publishers, 28 & 29, Southampton Street, London, W.C.

